# Supplementary material for: Resolving the full spectrum of human genome variation using Linked-Reads
Source: Genome Res. 2019 Apr;29(4):635–45. doi: 10.1101/gr.234443.118 (PMC6442396; doi:10.1101/gr.234443.118)
Supplement: Supplemental Material [file supp_gr.234443.118_Supplemental_longranger-2.2.zip › longranger-2.2.tar.bz2!/lr2.2-public/lib/stan/cmdstan/stan/doc/api/html/rev_2functions_2erf_8hpp_source.html]

Stan: src/stan/agrad/rev/functions/erf.hpp Source File


|  |  |
| --- | --- |
|  | Stan  2.5.0  probability, sampling & optimization |


- Main Page
- Related Pages
- Namespaces
- Classes
- Files

- File List
- File Members

All Classes Namespaces Files Functions Variables Typedefs Enumerations Enumerator Friends Macros Pages

- src
- stan
- agrad
- rev
- functions

erf.hpp

Go to the documentation of this file.

1 #ifndef STAN\_\_AGRAD\_\_REV\_\_FUNCTIONS\_\_ERF\_HPP

2 #define STAN\_\_AGRAD\_\_REV\_\_FUNCTIONS\_\_ERF\_HPP

3

4 #include <valarray>

5 #include <math.h>

6 #include <stan/agrad/rev/var.hpp>

7 #include <stan/agrad/rev/internal/v\_vari.hpp>

8 #include <stan/math/constants.hpp>

9

10 namespace stan {

11  namespace agrad {

12

13  namespace {

14  class erf\_vari : public op\_v\_vari {

15  public:

16  erf\_vari(vari\* avi) :

17  op\_v\_vari(::erf(avi->val\_),avi) {

18  }

19  void chain() {

20  avi\_->adj\_ += adj\_ \* stan::math::TWO\_OVER\_SQRT\_PI

21  \* ::exp(- avi\_->val\_ \* avi\_->val\_);

22  }

23  };

24  }

25

63  inline var erf(const stan::agrad::var& a) {

64  return var(new erf\_vari(a.vi\_));

65  }

66

67  }

68 }

69 #endif

stan::agrad::erf

fvar< T > erf(const fvar< T > &x)

**Definition:** erf.hpp:17

stan::agrad::var::vi\_

vari \* vi\_

Pointer to the implementation of this variable.

**Definition:** var.hpp:40

stan::math::TWO\_OVER\_SQRT\_PI

const double TWO\_OVER\_SQRT\_PI

**Definition:** constants.hpp:146

v\_vari.hpp

var.hpp

stan::agrad::var

Independent (input) and dependent (output) variables for gradients.

**Definition:** var.hpp:27

constants.hpp

stan::agrad::exp

fvar< T > exp(const fvar< T > &x)

**Definition:** exp.hpp:16


---

[ Stan Home Page ]

*© 2011–2014,
Stan Development Team.*
